# Quantum Dots as Multifunctional Materials for Tumor Imaging and Therapy

**DOI:** 10.3390/ma6020483

**Published:** 2013-02-05

**Authors:** Longfei Liu, Qingqing Miao, Gaolin Liang

**Affiliations:** CAS Key Laboratory of Soft Matter Chemistry, Department of Chemistry, University of Science and Technology of China, 96 Jinzhai Road, Hefei, Anhui 230026, China; E-Mails: llftt2@mail.ustc.edu.cn (L.L.); qqmiao@mail.ustc.edu.cn (Q.M.)

**Keywords:** quantum dots, tumor imaging and therapy, semiconductor, fluorescence, probe

## Abstract

The rapidly developing field of quantum dots (QDs) provides researchers with more options for imaging modalities and therapeutic strategies. In recent years, QDs were widely used as multifunctional materials for tumor imaging and therapy due to their characteristic properties such as semiconductive, zero-dimension and strong fluorescence. Nevertheless, there still exist the challenges of employing these properties of QDs for clinical diagnosis and therapy. Herein, we briefly review the development, properties and applications of QDs in tumor imaging and therapy. Future perspectives in these areas are also proposed as well.

## 1. Introduction

### 1.1. Quantum Dots

**Figure 1:**
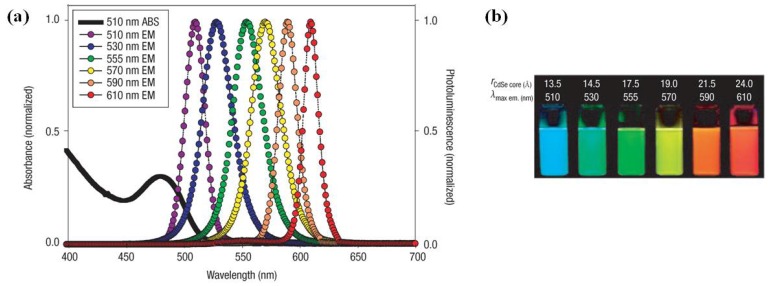
(**a**) Absorption and emission of six different quantum dot (QD) dispersions. The black line shows the absorption of the 510 nm emitting QDs. Note that at the wavelength of lowest absorption for the 510 nm QD, ~450 nm, the molar extinction coefficient is greater than that of rhodamine red at its absorption maxima (~150,000 *vs.* 129,000 M^−1^ cm^−1^); (**b**) Photo demonstrating the size-tunable fluorescence properties and spectral range of the six QD dispersions plotted in A *vs.* CdSe core size. All samples were excited at 365 nm with a UV source. For the 610 nm emitting QDs, this translates into a Stokes shift of ~250 nm. r = radius. Reprinted with permission from [5]. Copyright 2005 Nature Publishing Group.

The quantum dot (QD) is defined as an artificially structured system with the capacity to load electrons [1]. Its special physicochemical properties differentiate it from other naturally occurring biogenic and anthropogenic nanoparticles [2]. QDs are one type of nanoparticles (NPs) with three characteristic properties: semiconductors, zero-dimension, and strong fluorescence. Although colloidal semiconductor QDs are single crystals with diameters of a few nanometers, their sizes and shapes can be precisely controlled by the duration, temperature, and ligand molecules during the synthetic processes [3]. The well controlled synthetic process yields QDs with composition- and size-dependent absorption and emission. Generally, as the sizes of QDs are reduced, the electronic excitations shift to higher energies (*i.e.*, shorter wavelengths) [4], such as CdSe QDs (shown in Figure 1) [5]. Besides, physical properties of QDs influence their fluorescence emissions. The fluorescence emission of colloidal QDs on the surface of a two-dimensional slab of photonic crystals will be enhanced due to a combination of high-intensity near fields with strong coherent scattering, which is related to leaky eigenmodes of the photonic crystal (In QDs’ periodically modulated structures, an anomalous resonant phenomenon arises from periodic index modulation of the refractive index, which allows phase-matching of externally incident radiation into modes that can be re-radiated into free space). Owing to the fact that these modes possess finite lifetimes within such structures, they are called “leaky eigenmodes” [6]. Another example of the fluorescence enhancement of QDs is that when a sharp gold tip is brought within a few nanometers from a single QD cluster surface, the fluorescence of the QD in the vicinity of the tip increases about fourfold in magnitude [7].

The specific properties of QDs enable them with wide applications in chemistry, chemical biology and biomedicine. In the past decade, QDs have been broadly applied in fluorescence resonance energy transfer (FRET) analysis, gene technology, fluorescent labeling of cellular proteins, cell tracking, *in vivo* animal imaging and tumor biology investigation [8]. In this review, we will discuss the applications of QDs in tumor imaging and therapy.

### 1.2. Tumor Imaging

**Figure 2:**
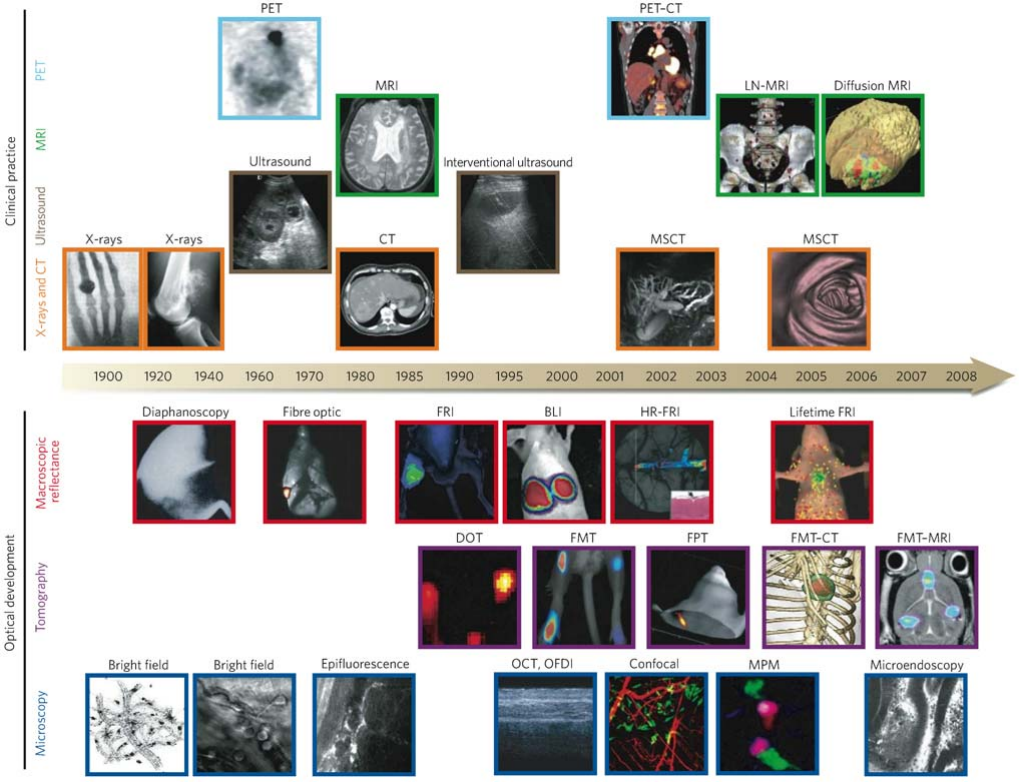
Imaging technologies used in oncology. Many macroscopic imaging technologies (shown above the timeline) are in routine clinical use, and there have been huge advances in their capabilities to obtain anatomical and physiological information since the beginning of the twentieth century. Shown are some examples of bones (X-rays), soft tissue (ultrasound, MRI and CT rows), three-dimensional organs (CT and MRI rows) and physiological imaging (MRI and PET rows). Microscopic and other intravital optical techniques (shown below the timeline) have developed over the past decade and now allow studies of genetic, molecular and cellular events *in vivo*. Shown are surface-weighted, whole-mouse, two-dimensional techniques (macroscopic reflectance row); tomographic three-dimensional techniques, often in combination with other anatomical modalities (tomography row); and intravital microscopy techniques (microscopy row). The timeline is approximate and is not to scale. Here BLI, bioluminescence imaging; CT, computed tomography; DOT, diffuse optical tomography; FMT, fluorescence-mediated tomography; FPT, fluorescence protein tomography; FRI, fluorescence reflectance imaging; HR-FRI, high-resolution FRI; LN-MRI, lymphotropic nanoparticle-enhanced MRI; MPM, multiphoton microscopy; MRI, magnetic resonance imaging; MSCT, multislice CT; OCT, optical coherence tomography; OFDI, optical frequency-domain imaging; PET, positron-emission tomography. Reprinted with permission from [9]. Copyright 2008 Nature Publishing Group.

Tumor imaging is an imaging technology, which monitors changes of tumor cells at tissue, cellular, or sub-cellular levels. It helps people with qualitative and quantitative analyses of the biological processes of tumors in imaging aspects. The three predominant imaging modalities are optical imaging (e.g., fluorescence or non-fluorescence imaging), nuclear imaging (e.g., single photon emission computed tomography (SPECT) and positron emission tomography (PET)), and Magnetic Resonance Imaging (MRI). Figure 2 and Table 1 show the development of imaging techniques and their characteristics, respectively [9]. Compared with other imaging methods, QDs have advantages of broad light spectrum emission from visible to infrared due to their controllable sizes, bright and photostable fluorescence within a few nanometers, and good water-solubility [10]. However, QDs have inherent disadvantages such as high cytotoxicity (e.g., QDs of CdSe) [10].

As one of the optical imaging modalities, fluorescence refers to relatively-longer-wavelength light (especially visible light) emitted by certain molecule after it absorbing light at a particular wavelength [9]. The fluorophores are classified into two groups in terms of their origin: Endogenous fluorophores and exogenous fluorophores. For example, NADH (reduced form of nicotinamide-adenine dinucleotide) is one type of endogenous fluorophores, which can indicate the metabolic status of tumor because only its reduced form has fluorescence [11]. Exogenous fluorophores are usually organic compounds with fluorogenic motifs (e.g., near-infrared (NIR) fluorophores such as heptamethine cyanines containing benzoxazole or benzothiazole motifs [12].

As the two main modalities for nuclear imaging, SPECT and PET play important roles of imaging bone metastases in miscellaneous cancers including lung cancer, thyroid cancer, renal cancer, myeloma, and neuroendocrine cancers [13]. While SPECT uses one photon with lower energy to produce three-dimensional image of tracer distribution with multiplanar images, PET uses positron-emitting radiotracers and achieves image with higher spatial resolution than that of SPECT [13]. MRI is also frequently applied in tumor imaging using dotarem or magnevist as T1 contrast media or resovist as T2 contrast medium [14]. To date, multimodalities of imaging such as PET-CT, PET-MRI have emerged and been used for the acquisition of images with higher accuracy [9].

QDs plays an increasingly important role in tumor imaging, especially near-infrared (NIR, 700–900 nm) imaging. NIR fluorescence imaging of tumor is expected to have a major impact in biomedical imaging because in the NIR region the absorbance spectra for all the biomolecules in tumor reach their minima, which provides a clear window for *in vivo* optical imaging of tumor [15]. In 2010, Gao *et al.* reported that QD800-MPA (a NIR non-cadmium QDs coated with mercaptopropionic acid with an emission wavelength of about 800 nm) had high tumor uptake and excellent contrast of tumor to surrounding tissues due to the enhanced permeability and retention (EPR) effect of this kind of ultrasmall nanoparticles [16].

**Table 1:** Overview of imaging systems. Reproduced with permission from [9]. Copyright 2008 Nature Publishing Group.

Technique	Resolution *	Depth	Time ^†^	Quantitative ^‡^	Multi-channel	Imaging agents	Target	Cost ^§^	Main small-animal use	Clinical use
MRI	10–100 μm	No limit	Minutes to hours	Yes	No	Paramagnetic chelates, magnetic particles	Anatomical, physiological, molecular	$$$	Versatile imaging modality with high soft-tissue contrast	Yes
CT	50 μm	No limit	Minutes	Yes	No	Iodinated molecules	Anatomical, physiological	$$	Imaging lungs and bone	Yes
Ultrasound	50 μm	cm	Seconds to minutes	Yes	No	Microbubbles	Anatomical, physiological	$$	Vascular and interventional imaging ^||^	Yes
PET	1–2 mm	No limit	Minutes to hours	Yes	No	^18^F-,^64^Cu- or ^11^C-labelled compounds	Physiological, molecular	$$$	Versatile imaging modality with many tracers	Yes
SPECT	1–2 mm	No limit	Minutes to hours	Yes	No	^99m^Tc- or ^111^In-labelled compounds	Physiological, molecular	$$	Imaging labelled antibodies, proteins and peptides	Yes
Fluorescence reflectance imaging	2–3 mm	<1 cm	Seconds to minutes	No	Yes	Photoproteins, fluorochromes	Physiological, molecular	$	Rapid screening of molecular events in surface-based disease	Yes
FMT	1 mm	<10 cm	Minutes to hours	Yes	Yes	Near-infrared fluorochromes	Physiological, molecular	$$	Quantitative imaging of fluorochrome reporters	In development
Bioluminescence imaging	Several mm	cm	Minutes	No	Yes	Luciferins	Molecular	$$	Gene expression, cell and bacterium tracking	No
Intravital microscopy ^¶^	1 μm	<400–800 μm	Seconds to hours	No	Yes	Photoproteins, fluorochromes	Anatomical, physiological, molecular	$$$	All of the above at higher resolutions but limited depths and coverage	In development ^#^

* For high-resolution, small-animal imaging systems. (Clinical imaging systems differ.); ^†^ Time for image acquisition; ^‡^ Quantitative here means inherently quantitative. All approaches allow relative quantification; ^§^ Cost is based on purchase price of imaging systems in the United States: $, <US$100,000; $$, US$100,000–300,000; $$$, >US$300,000; ^||^ Interventional means used for interventional procedures such as biopsies or injection of cells under ultrasound guidance; ^¶^ Laser-scanning confocal or multiphoton microscopy; ^#^ For microendoscopy and skin imaging.

### 1.3. Tumor Therapy

Millions of people die from cancer every year, especially from lung cancer. Even though no existing method can defeat cancer, tumor therapies such as surgery, radiotherapy, chemotherapy, and photodynamic therapy (PDT) are developing dramatically nowadays. Surgery has relatively good effects for benign tumors and precancerous tumors. Advances in radiotherapy, such as intensity-modulated radiation therapy (IMRT), provide the capability of delivering a highly conformal distribution of radiative dose to a static, complex targeting volume [17]. Chemotherapy is widely adopted for tumor therapy and hundreds of anticancer drugs are used clinically. Typical anticancer drugs include plant extractives (e.g., taxol [18]), heavy metal complexes (e.g., cisplatin [19]), bioreductive drugs (e.g., tirapazamine, 3-amino-1,2,4-benzotriazine 1,4-dioxide or TPZ [20]), and traditional Chinese medicine [21]. In *bcl*-2-positive cancer cells (cancer cells expressing *bcl*-2), taxol induces the phosphorylation of *bcl*-2 and programmed cell death thereafter [22]. Cisplatin, one of the most widely used anticancer drugs, can bind with DNA to form *cis*-DDP/DNA adducts which induce DNA-damage and cell apoptosis [19]. Tirapazamine (TPZ), a leading bioreductive drug with selective cytotoxicity to hypoxic cells in tumor, damages the DNA inside cell with the reactive oxygen species (ROS) with its reduction product under the action of reductases in cells [20].

PDT has been an innovative and attractive modality for treatment of small and superficial tumors since the end of the last century [23]. After absorption of light with certain wavelength, sensitizers can induce the necrosis of tumors [23]. Some examples of QDs for PDT of tumors are discussed in the following parts.

## 2. QDs for Tumor Imaging

QDs, tiny light-emitting particles on nanometer scale, are new type of fluorescent probes for molecular and cellular imaging. Compared with organic dyes and fluorescent proteins, QDs have unique optical and electronic properties in cellular imaging: Wavelength-tunable emission, improved brightness of signal, resistance against photobleaching, *etc*. [24]. Such preponderant optical properties were not realized until the QD-based probes are equipped with war heads targeting tumor. Xingyong Wu and co-workers synthesized immunofluorescent probes by conjugating the QDs with streptavidin or IgGs (immunoglobulin Gs). Using the conjugates, they conducted comprehensive investigations on cell imaging at the targets of interest including cell surface receptors, cytoskeleton components, and nuclear antigens [25]. Up to date, QDs have been rapidly developed in tumor imaging, such as imaging tumor vasculature [15] and sentinel lymph node [26].

### 2.1. QDs for Imaging Membrane Receptors (Surface)

**Figure 3:**
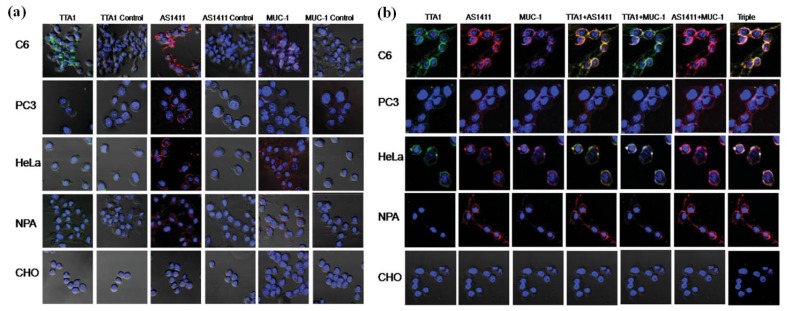
Confocal microscopy imaging of cells treated with QD-aptamer conjugates. (**a**) A single incubation of QD605-TTA1, QD655-AS1411, or QD705-MUC-1 was applied to PC-3, HeLa, CHO, C6, and NPA cells, and confocal images were obtained. Each image was compared with the corresponding QD-control aptamers (column 1: QD-TTA1, column 2: QD-TTA1 control, column 3: QD-AS1411, column 4: QD-AS1411 control, column 5: QD-MUC-1, column 6: QDMUC- 1control); (**b**) Multiplex imaging of cancer cells treated simultaneously with three different types of QD-conjugated aptamers. Single images for QD-TTA1 (605 nm, light green, column 1), QD-AS1411 (655 nm, red, column 2), and QD-MUC-1(705 nm, violet, column3), dual images for QD-AS1411 and QD-TTA1 (column 4, yellow for co-localization), QD-TTA1 and QD-MUC-1 (column 5, light green for co-localization), and QD-AS1411 and QD-MUC-1 (column 6, violet for co-localization), and a triple image for QD-AS1411, QD-TTA1, and QD-MUC-1 (column 7, white for co-localization) were acquired from PC-3, HeLa, CHO, C6, and NPA cells. All figures are merged with the 40, 6-diamidino-2-phenylindole (DAPI) image (nucleus staining, 460 nm) and cellular morphology. Reprinted with permission from [29]. Copyright 2009 John Wiley and Sons.

Metastases, which are responsible for most cancer deaths rather than those of primary tumors, spread tumor cells from a primary site to new distant organs [27]. Changes of membrane morphology or dynamics of membrane protein in cancer cells for cellular fluidity are critical for cancer metastasis [28]. Therefore, QDs modified with targeting ligands offer a good opportunity to track the changes of related membrane receptors. Soonhag Kim and co-workers designed a series of aptamers conjugating with different QDs to image the proteins on the membranes of cancer cells [29]. They chose three different QDs with distinct emission wavelengths of 605, 655 and 705 nm to respectively conjugate with three cancer-related aptamers-AS1411, TTA1, and MUC-1 [29]. AS1411 is an aptamer that binds to the nucleolin in the plasma membranes of cancer cells [30,31]. TTA1, which is expressed during the tissue remodeling processes including angiogenesis, inflammation, and tumor growth, binds to the extracellular matrix protein tenascin-C of cancer cells [32]. MUC-1 targets mucin, which is highly expressed by the majorities of human adenocarcinomas [33,34]. Confocal microscopic cell images were obtained with the receptors in different cells being successfully labeled with respective QD-conjugated aptamers (shown in Figure 3) [29]. Gonda *et al.* [28] also labeled a metastasis-promoting factor on the cell membrane called protease-activated receptor 1 (PAR1) with QD-conjugated anti-PAR1 antibody. By tracking the fluorescence of QDs, they photographed four stages of metastasis: cancer cells far from blood vessels in tumor, near the vessel, in the bloodstream, and adherent to the inner vascular surface in the normal tissues near tumor. With this, they successfully showed the dynamics of PAR1 movement in the whole process [28].

### 2.2. QDs for Imaging Cytoskeleton Components (Intracellular)

The cellular cytoskeleton, involved in many fundamental processes (e.g., locomotion and cytokinesis) of the cell, consists of actin filaments, microtubules and intermediate filaments [35]. Tumor cells are in endless division, which is related to the movement of actin filaments and microtubules [36,37]. Therefore, imaging the movement of actin filaments and microtubules in tumor cells is important for tumor imaging.

**Figure 4:**
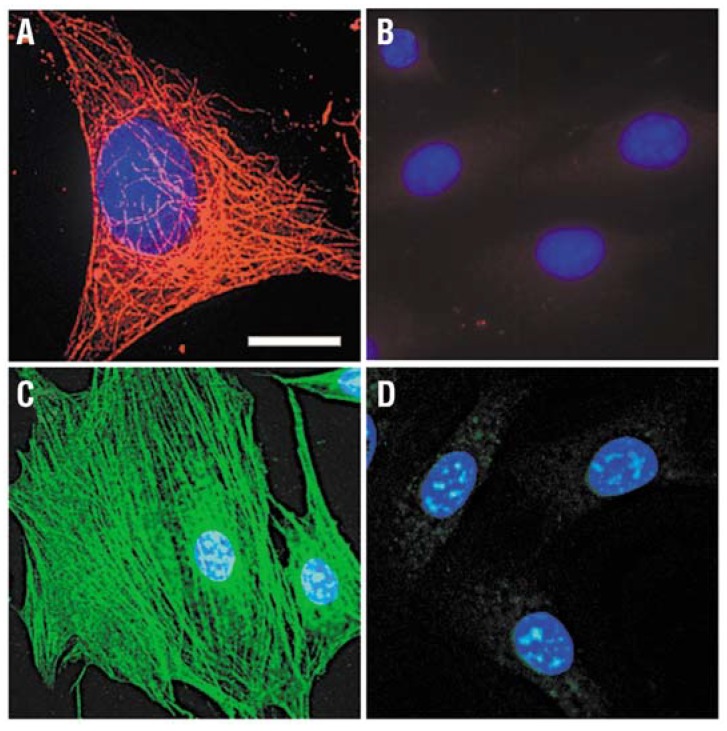
Staining of cytoskeleton fibers in 3T3 mouse fibroblast cells with QD-streptavidin. (**A**) Microtubules were labeled with monoclonal anti-α-tubulin antibody, biotinylated anti-mouse IgG and QD 630-streptavidin (red); (**B**) Control for (**A**) without primary antibody; (**C**) Actin filaments were stained with biotinylated phalloidin and QD 535-streptavidin (green); (**D**) Control for (**C**) without biotin-phalloidin. The nuclei were counterstained with Hoechst 33342 blue dye. Filter sets ex. 480 ± 20 nm/em. 535 ± 10 nm and ex. 560 ± 27.5 nm/em. 635 ± 10 nm were used to observe signals of QD 535 and QD 630, respectively. Scale bar, 10 μm for (**A**), 24 μm for (**B**) through (**D**). Reprinted with permission from [25]. Copyright 2003 Nature Publishing Group.

In the end of the last century, Bruchez *et al.* labeled the F-actin filaments with red nanocrystal probes conjugated with biotin [38]. Compared with conventional dye molecules, the nanocrystal-labeled samples showed advantages of neglectable photobleaching [38]. Wu *et al.* used QD 630-streptavidin (red) and QD 535-streptavidin (green) to stain microtubules and actin filaments, respectively [25]. The results indicated the QD-based probes could be bright enough and specific enough for effectively labeling fine cellular structures, and have a better performance over other probes reported (shown in Figure 4) [25]. In 2008, Higuchi *et al.* reported new photostable, bright QDs conjugated with anti-tubulin antibody, which could bind to microtubules and trace the dynamic movement of microtubules in living cancer cells [39].

### 2.3. QDs for Imaging Nuclear Antigens (Intranuclear)

**Figure 5:**
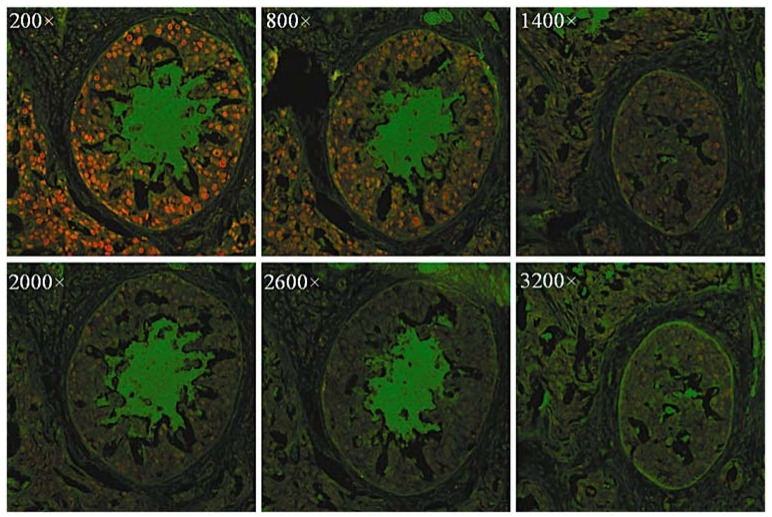
Fluorescence images of breast cancer tissues labeled with CdSe/ZnS QDs. PCNA was stained red with QDs modified with the antibody. The original QD-SA concentration was about 1 μmol/L and was diluted 200–3200× before staining operations. Reprinted with permission from [40]. Copyright 2010 Society for Applied Spectroscopy.

Tumor cells have some specific over-expressed nuclear antigens relating to their endless proliferation, such as PCNA (proliferating cell nuclear antigen) [40]. It was reported that QDs coated with urea or acetate groups might stain the nucleus [38]. Tang *et al.* used CdSe/ZnS QDs conjugated with anti-human PCNA antibody to label PCNA (proliferating cell nuclear antigens) in breast cancer tissues (shown in Figure 5) [40]. Xingyong Wu used QD 630-streptavidin to label the nuclei of SK-BR-3 cells successfully [25]. Labeling nuclear antigens in tumor cells with QD-conjugated bioprobes offers people with useful and reliable information for biomedical analysis and cancer diagnosis.

### 2.4. QDs for Imaging Tumor Neovasculature (a Special Example of Tumor Imaging)

**Figure 6:**
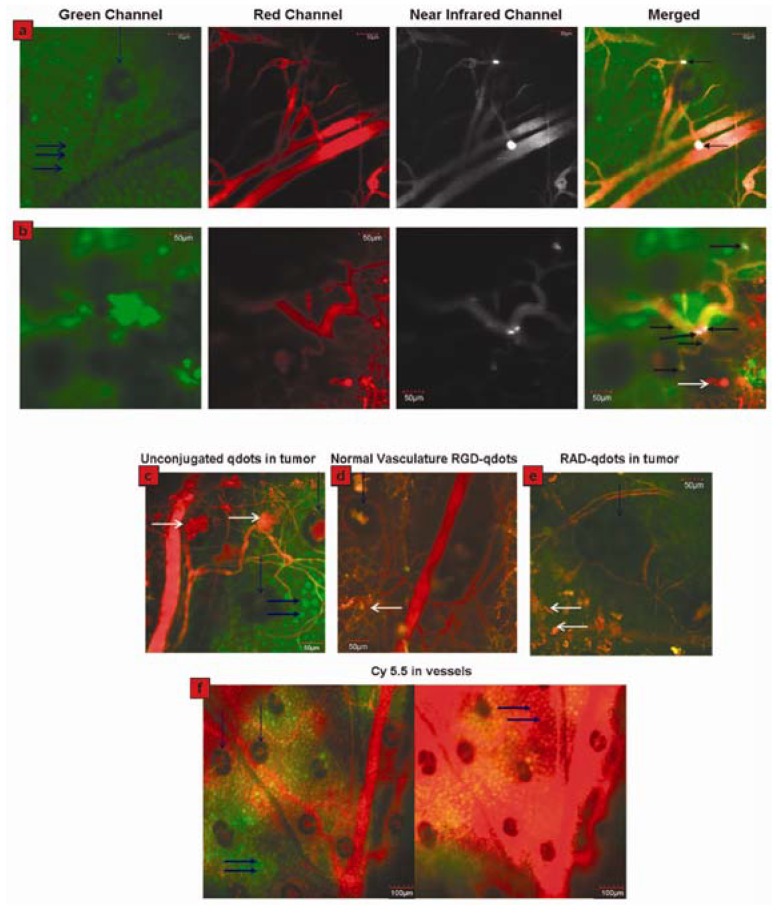
Direct visualization of binding of RGD-QDs to tumor vessel endothelium and controls. (**a**) Panel displays different output channels of the identical imaging plane along the row with scale bars. In the green channel, individual EGFP-expressing cancer cells are visible (marked by thick horizontal blue arrows; vertical blue arrow points to a hair follicle), while the red channel outlines the tumor’s vasculature *via* injection of Angiosense dye. The NIR channel shows intravascularly administered QDs, which remain in the vessels (*i.e.*, they do not extravasate). Binding events are visible by reference to bright white signal. These are demarcated by arrows in the rightmost merged image, in which all three channels have been overlaid; (**b**) Displays the same as (**a**) in a different mouse, except that six times the RGD-QDs dose has been injected. Individual cells are not generally visible. Six binding events are observed in this FOV, as marked by arrows in the merged image at right. White arrows in the bottom merged image designate areas of tissue autofluorescence. Typical images of no binding in each control condition are shown in (**c**–**f**). Tumor neovasculature containing unconjugated QDs (**c**), normal vasculature containing RGD-QDs (**d**), and tumor neovasculature containing RAD-QDs (**e**). (**f**) Tumor vasculature shortly after Cy5.5 injection (left) and ~20 min post-Cy5.5 injection (right). Individual cancer cells are visible before (left) and after dye extravasates (right, dyed red). Also see movie S6 in Supporting Information. Horizontal white arrows indicate tissue autofluorescence, vertical blue arrows denote hair follicles (which generally display autofluorescence in their center), and thick horizontal blue arrows indicate individual cancer cells. Reprinted with permission from [41]. Copyright 2008 American Chemical Society.

Newly formed/forming blood vessels express α_v_β_3_ integrin, which specifically binds to arginine-glycine-aspartic (RGD) peptides. The α_v_β_3_ integrin receptor plays an important role in tumor metastasis and tumor-induced angiogenesis, making it possible for RGD-conjugated QDs to image tumor neovasculature [41]. Employing this, Sanjiv Sam Gambhir and co-workers designed RGD-QDs for real-time intravital imaging of luminal endothelium in mouse tumor neovasculature [41]. The peptides for QDs conjugation are cyclo(RGDfC) and cyclo(RADfC). In contrast to the controls, RGD-QDs specifically bound to tumor vessel endothelium and exhibited better performance than organic dyes (shown in Figure 6). Importantly, this work of real-time imaging tumor neovasculature was performed in living subjects with an intravital microscopy, which opens the door of *in vivo* tumor imaging with QDs.

## 3. QDs for Tumor Therapy

**Figure 7:**
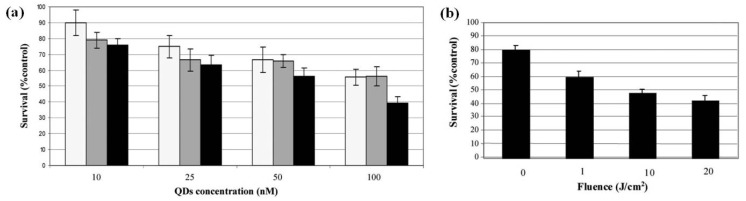
(**a**) Measurement of PDT sensitivity of KB cells treated with FA-conjugated **QD 4**. Cells were exposed to QDs in a concentration range of 10–100 nM for 1 (light gray), 3 (dark grey) and 6 h (black); (**b**) Survival curves obtained for cells incubated with QDs at 5 nM for 3 h incubation before irradiation to increasing doses of light from 1 to 20 J cm^−2^. Measurement of PDT sensitivity for the QDs were obtained by MTT test (data points show the mean ± s.d., *n* = 6). * P < 0.05 *vs.* previous fluence dose. Reprinted with permission from [45]. Copyright 2011 Royal Society of Chemistry.

Although relatively fewer researches on QDs for tumor therapy were reported, it is conceivable that QDs have the potentialities for tumor therapy due to their large surface areas available for the modification of functional groups or therapeutic agents such as anti-cancer drugs [42] and PDT photosensitizers (PS) [43]. Moreover, QDs themselves can also functionalize as PDT photosensitizers for tumor therapy [44]. Herein, QDs as PDT photosensitizers and anti-cancer drug-QD complexes for cancer therapy are reviewed as following. Barberi-Heyo *et al.* established that QDs conjugated with folic acid (FA) could be used as PS for PDT of cancer [45]. They conjugated CdTe(S)-type QDs with FA which is an optimal targeting ligand for selectively delivering the attached therapeutic agents (herein QDs as PS) to cancer tissues. 3-(4,5-dimethylthiazol-2-yl) 2,5 diphenyl tetrazolium bromide (MTT) assay indicated that the survival rate of KB (human head and neck carcinoma cell line) cells incubated with FA-conjugated QDs decreased as the irradiation time or intensity increased (shown in Figure 7). The results demonstrated that CdTe(S)-type QDs had photosensitizing properties, which could be used to promote PDT effect. In their study, they mentioned that the concentration of QDs should be inferior to 10 nM and the incubation time less than 8 hour to avoid the intrinsic cytotoxicity of QDs without light irradiation.

**Figure 8:**
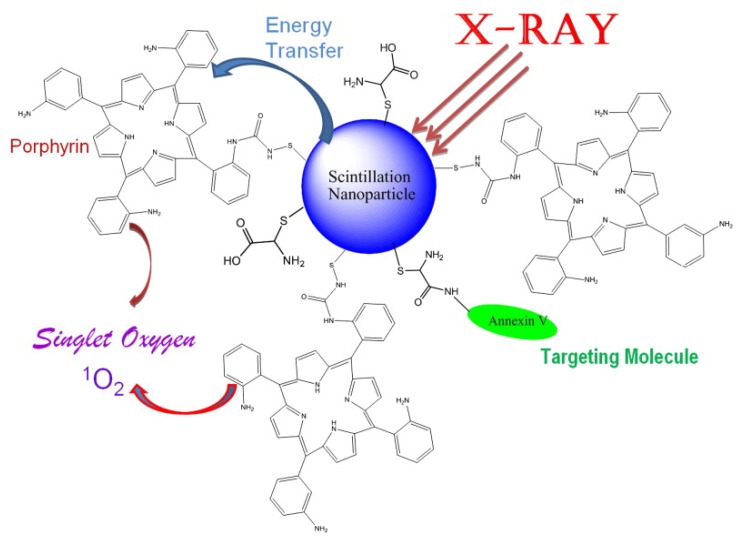
Schematic presentation of the nanoparticle-based X-ray-induced PDT. Under ionizing radiation a nanoparticle starts to scintillate transferring its energy into a conjugated porphyrin molecule, which then generates singlet oxygen necessary to produce photosensitizing effect. This methodology will help to treat nodular and deeper tumors due to higher penetrating capacity of X-rays and gamma rays compared to that of visible light commonly used in PDT. Reprinted with permission from [43]. Copyright 2008 Elsevier.

**Figure 9:**
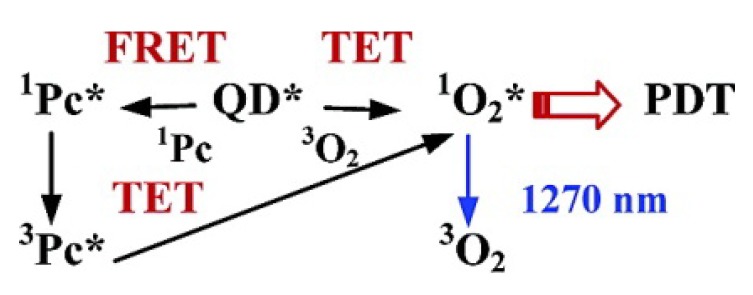
Schematics of the ^1^O_2_ generation in QD-based PDT systems. Reprinted with permission from [46]. Copyright 2003 American Chemical Society.

Taking advantage of QDs’ superior physical properties, PS-QDs conjugates can be excited with a wide range of wavelengths and avoid the PS to absorb light in the mean time (Figure 8 explains how QDs assist photosensitizers with producing singlet oxygen) [43]. Clemens Burda and co-workers used QD-based fluorescence resonance energy transfer (FRET) to facilitate the excitation of a PDT photosensitizer to generate reactive ^1^O_2_ species for PDT [46]. The results demonstrated that CdSe QDs could be used to sensitize either phthalocyanines (a family of PDT agents) such as Pc4 *via* FRET mechanism or itself as PS *via* a triplet energy transfer (TET) mechanism to produce ^1^O_2_ species for PDT (shown in Figure 9).

## 4. Multifunctional QDs for Synchronous Tumor Imaging and Therapy

**Figure 10:**
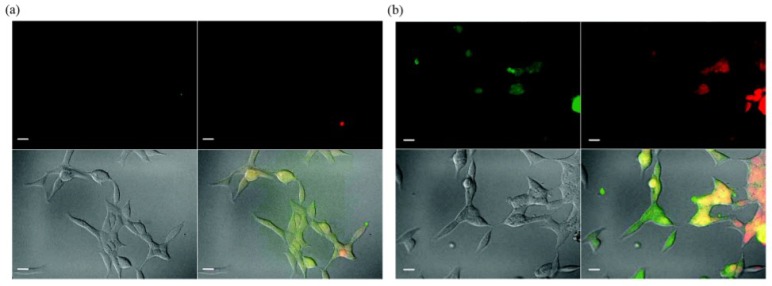
Confocal laser scanning microscopy images of PSMA expressing LNCaP cells after incubation with 100 nM QD-Apt-(Dox) conjugates for 0.5 h at 37 °C, washed two times with PBS buffer, and further incubated at 37 °C for (**a**) 0 h and (**b**) 1.5 h. Dox and QD are shown in red and green, respectively, and the lower right images of each panel represents the overlay of Dox and QD fluorescent. The scale bar is 20 μm. Reprinted with permission from [47]. Copyright 2007 American Chemical Society.

**Figure 11:**
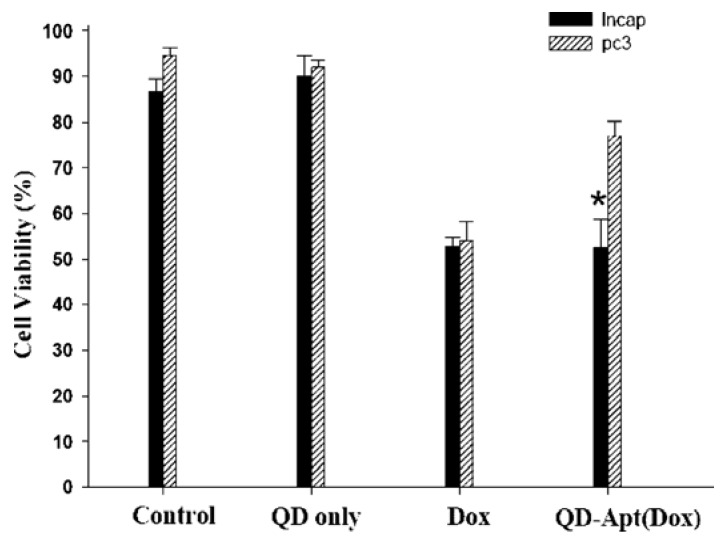
Growth inhibition assay (MTT). Prostate cancer cell lines, LNCaP (PSMA+) and PC3 (PSMA−), were incubated with QD alone (1.6 μM), Dox along (5 μM), or QD-Apt(Dox) conjugates (1.6 μM), for 3 h, and the cells were washed and further incubated for 24 h prior to measurement of cell viability. Asterisk indicates significant differences between LNCaP and PC3 cells, (P < 0.005, *n* = 3). Reprinted with permission from [47]. Copyright 2007 American Chemical Society.

QDs have exhibited specific advantages in tumor imaging and tumor therapy. Large surface area of QDs enables them to be conjugated with different agents including targeting molecules, therapeutic chemicals, and imaging substances. Obviously, the multifunctional QDs for synchronous tumor imaging and therapy will be much more attractive and important. Sangyong Jon and co-workers reported such a multifunctional QD-aptamer (Apt)-doxorubicin (Dox) conjugate [QD-Apt(Dox)] for cancer-targeted imaging, therapy, and sensing [47]. The conjugate consists of three components: QDs, which functionalize as fluorescent agents; RNA aptamers covalently attached to the surface of QD, which serve a dual functions as targeting molecules and as drug carrying vehicles; Dox, which is a therapeutic agent for tumor cells as well as a fluorescent agent (Dox has fluorescence itself [48]). This conjugate keeps fluorescence-off state through a Bi-FRET mechanism when Dox links to QD. Fluorescence of both QD and Dox will be turned on after Dox being released from the QD-conjugate. The results indicated that QD-Apt(Dox) could differentially bind to prostate specific membrane antigen(PSMA)-expressing LNCaP cells instead of the PSMA-negative PC3 prostate adenocarcinoma cell lines due to the aptamer selectively binding to PSMA. Fluorescence microscopic cell imaging indicated that Dox was released from QD-conjugate 1.5 h after endocytosis and the targeting cells were stained with both Dox and QD (shown in Figure 10). MTT assay indicated that the QD-Apt(Dox) has LNCaP cell-targeted therapeutic ability (shown in Figure 11). 

In summary, this conjugate can be used to detect cancer cells at a single cell level. It exhibits specificity and sensitivity to LNCaP cells for sensing, imaging, and therapy.

## 5. Conclusions

Quantum Dots, as one type of multifunctional materials, have shown promising advantages in tumor imaging and therapy due to their specific physicochemical properties. Nevertheless, they still have some non-neglectable limitations such as increased sizes after coating [49] and the cytotoxicities introduced [2,50]. As we reviewed above, some QDs have impressive effect of imaging tumor neovasculature, which is however too late for clinical diagnosis of cancer development. These call for the development of new types of QDs for the detection of important biomarkers (e.g., furin) of cancers at early stages [51]. Development of QDs in the future will not be limited to tumor imaging or therapy, but could be a combination of two or multiple functions. We envision that QDs will become one type of promising material for real-time tumor-targeted imaging and therapy in the future.
